# Safety of CMR in patients with cardiac implanted electronic devices

**DOI:** 10.1186/1532-429X-18-S1-O123

**Published:** 2016-01-27

**Authors:** El-Sayed H Ibrahim, Laura Horwood, Jadranka Stojanovska, Anil Attili, Luba Frank, Hakan Oral, Frank Bogun

**Affiliations:** 1 University of Michigan, Ann Arbor, MI USA; 2 University of Texas Medical Branch, Galveston, TX USA

## Background

CMR has been reported to be safe in patients with cardiac implantable electronic device (CIED), provided a specific protocol is followed. The goal of this study is to examine whether this is true for patients excluded from published protocols, e.g. CIED patients with abandoned leads or pacemaker dependency.

## Methods

The protocol followed at our institution for MR imaging of CIED patients is as follows: 1) Necessity and absence of an alternative imaging modality; 2) Device-related relative contraindications, including the presence of abandoned leads, pacemaker dependency, and time to lead implant < 6 weeks, with the possibility of the ordering physician to overrule these exclusions. 3) A provider with CIED management expertise assesses the baseline device information, where the device was programmed according to the patient's needs with tachyarrhythmia detection and therapy disabled during the scan. 4) Device reinterrogation and reprogramming after completion of the scan, as well as at 1 week and 3 months after imaging.

## Results

**Figure 1:**
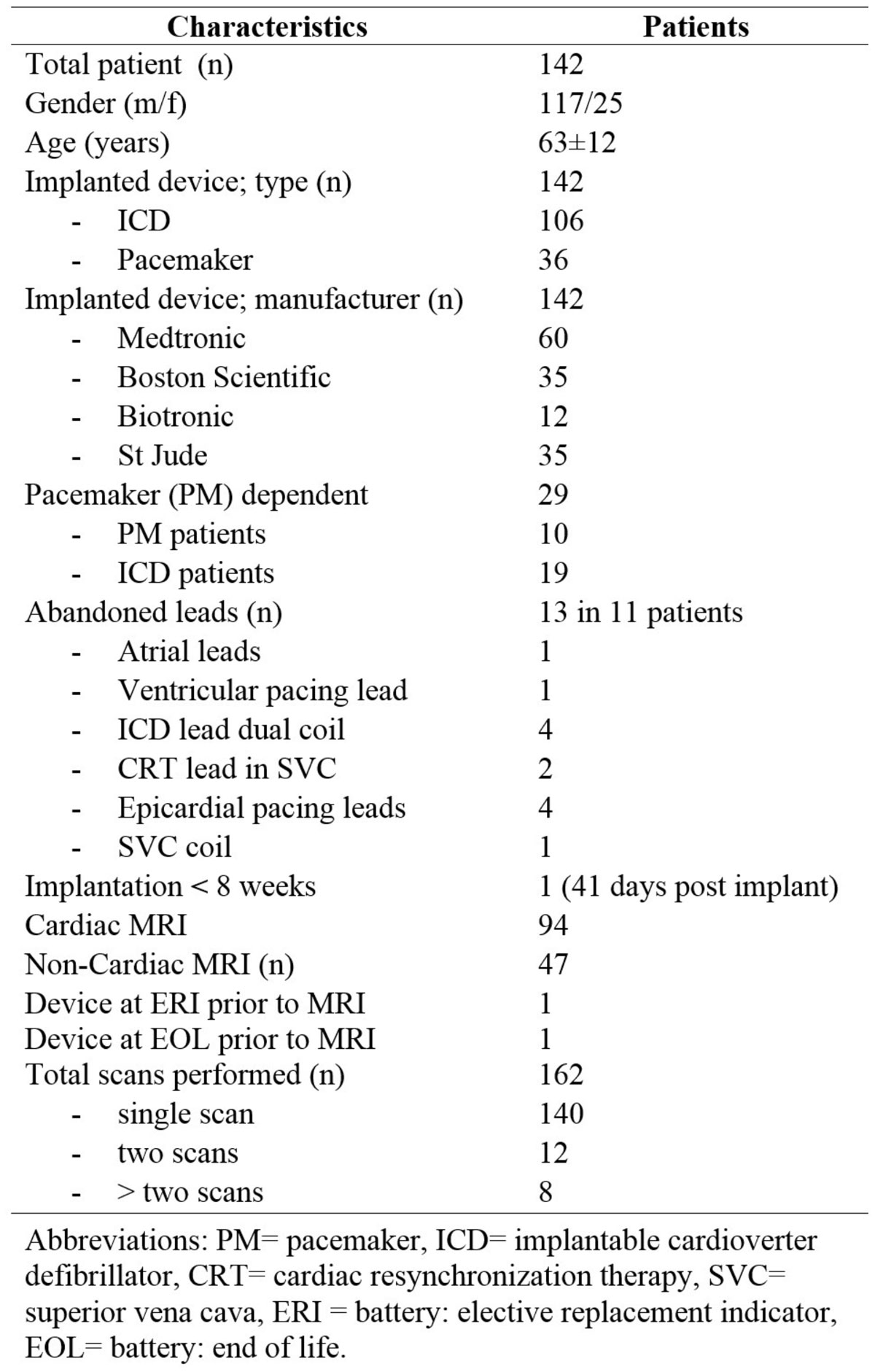
**Patients' characteristics**.

**Figure 2:**
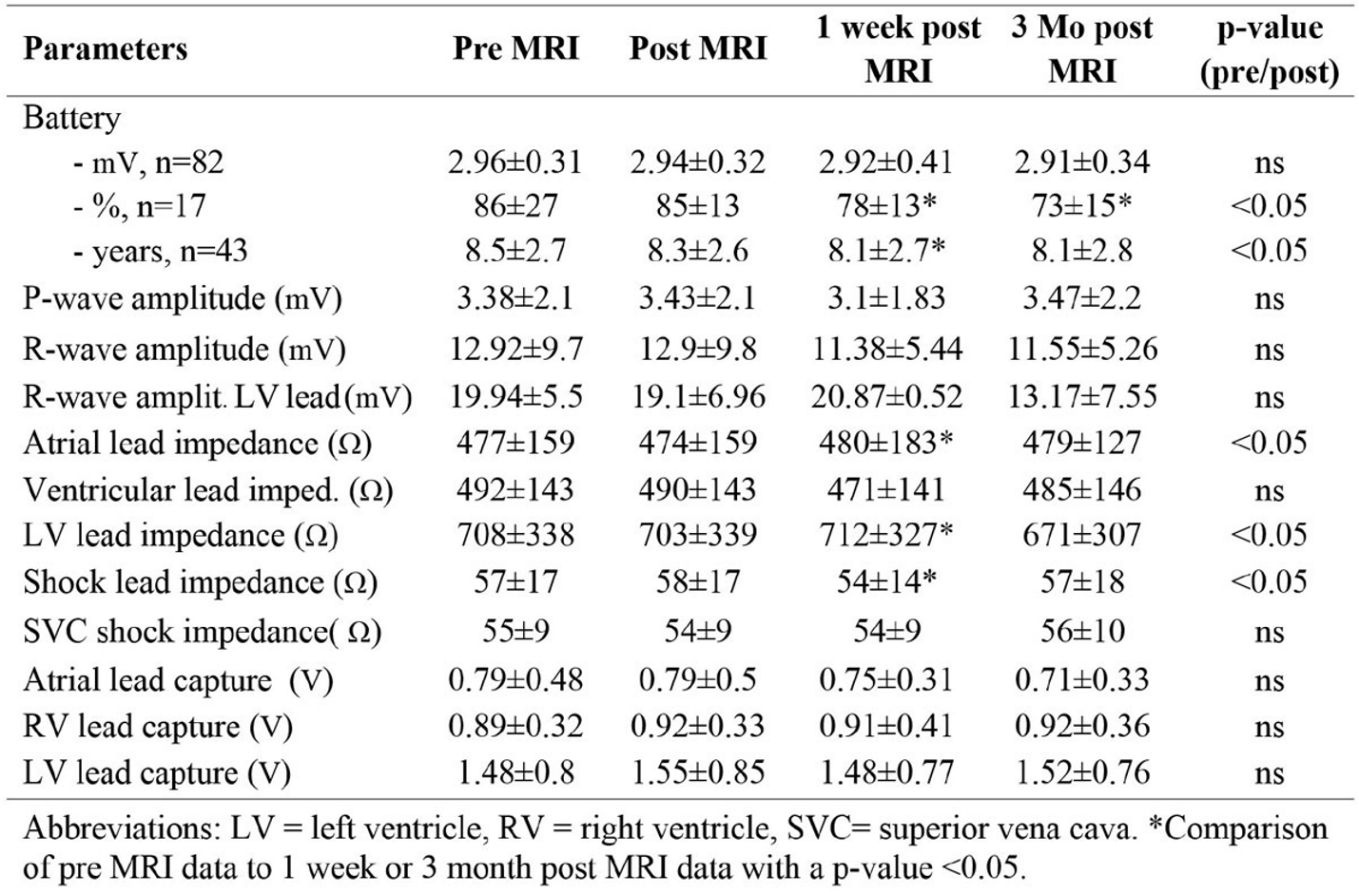
**Pacemaker and ICD data prior and post MR imaging**.

A total of 162 MR scans were obtained in 142 consecutive patients with CIED's (106 patients had defibrillators and 36 had pacemakers, as shown in Figure 1). 29 patients were pacemaker dependent and 11 patients had abandoned leads. Cardiac MR scans were performed in 94 patients (late gadolinium enhancement (LGE) was used to determine myocardial scar prior to ablation) and spinal/brain scans were performed in 47 patients. In the cardiac scans, the images were non-diagnostic only in 4 patients due to extensive artifact from the implanted cardiac defibrillator (ICD). In 65 patients, LGE was detected without artifact. No LGE was identified in 25 scans. Only one patient developed ventricular tachycardia (VT) during a spine scan and was removed from the scanner for device reactivation, which terminated VT without consequences. No other adverse events were noted. The device parameters essentially remained the same immediately, 1 week, and 3 months after the scans (Figure 2).

## Conclusions

In the analyzed cohort of 142 patients, only in one pacemaker dependent patient, the heart rate dropped from 90 to 50 bpm during spinal imaging using a fast spin echo sequence (specific absorption rate (SAR) = 1.89 W/Kg) due to elevated noise rate of the ICD. In this patient, a prior cardiac scan was performed a week earlier where no problem was observed, as the cardiac LGE sequences have low SAR of 0.11 W/Kg. Therefore, with the protocol described in this study, CMR imaging can be safely performed in CIED patients without exposing the patients to risk, despite pacemaker dependency, presence of abandoned leads, and other CIED contraindications. This study recommends that CMR imaging be available to more CIED patients who can benefit from it.

